# Primary syphilis cases in Guangdong Province 1995-2008: Opportunities for linking syphilis control and regional development

**DOI:** 10.1186/1471-2458-10-793

**Published:** 2010-12-30

**Authors:** Li-Gang Yang, Joseph D Tucker, Bin Yang, Song-Ying Shen, Xi-Feng Sun, Yong-Feng Chen, Xiang-Sheng Chen

**Affiliations:** 1 Guangdong Provincial Center for Skin Disease & STI Control, Guangzhou, PR China; 2 Division of Infection Diseases, Massachusetts General Hospital, Boston, USA; 3 National STD Control Center, Chinese Academy of Medical Sciences, Peking Union Medical College Institute of Dermatology, Nanjing, PR China

## Abstract

**Background:**

Syphilis cases have risen in many parts of China, with developed regions reporting the greatest share of cases. Since syphilis increases in these areas are likely driven by both increased screening and changes in sexual behaviours, distinguishing between these two factors is important. Examining municipal-level primary syphilis cases with spatial analysis allows a more direct understanding of changing sexual behaviours at a more policy-relevant level.

**Methods:**

In this study we examined all reported primary syphilis cases from Guangdong Province, a southern province in China, since the disease was first incorporated into the mandatory reporting system in 1995. Spatial autocorrelation statistics were used to correlate municipal-level clustering of reported primary syphilis cases and gross domestic product (GDP).

**Results:**

A total of 52,036 primary syphilis cases were reported over the period 1995-2008, and the primary syphilis cases increased from 0.88 per 100,000 population in 1995 to 7.61 per 100,000 in 2008. The Pearl River Delta region has a disproportionate share (44.7%) of syphilis cases compared to other regions. Syphilis cases were spatially clustered (p = 0.01) and Moran's I analysis found that syphilis cases were clustered in municipalities with higher GDP (p = 0.004).

**Conclusions:**

Primary syphilis cases continue to increase in Guangdong Province, especially in the Pearl River Delta region. Considering the economic impact of syphilis and its tendency to spatially cluster, expanded syphilis testing in specific municipalities and further investigating the costs and benefits of syphilis screening are critical next steps.

## Background

A commonly used term for syphilis infection in Southern China is "Guangdong boils", thought to be related to the historical position of Guangdong as an important economic and trade hub that has concomitantly suffered from substantial syphilis epidemics in the past. After nearly being eliminated in the 1950 s, syphilis has returned to Southern China, disproportionately affecting provinces with a higher gross domestic product (GDP) [1-3]. Syphilis is now the second most commonly reported communicable disease in Guangdong Province. A study in southern Guangdong Province among 477,656 pregnant women screened for syphilis found 0.5% had a positive treponemal test [4]. Cross-sectional epidemiology studies among MSM in Guangdong Province have found a high prevalence of both syphilis [5,6] and HIV infection [5,7].

Syphilis spread is a classic example of how some sexually transmitted infections are spatially clustered, making spatial analysis particularly useful for epidemiological analysis and targeted control programs [8]. Several studies suggest that syphilis cases cluster in specific geographic areas [8-10], but studies have generally been limited to high-income areas where the burden of syphilis is less than South China. Cross-sectional syphilis investigations and government surveillance data in China reveal that more economically developed provinces have a higher burden of syphilis [1,2], but there is less understanding at the municipal-level and none of the previous work has focused on primary syphilis cases that are likely a more sensitive indicator of changes in sexual behaviour.

Guangdong Province experienced very fast economic growth after China's "Open Door Policy" in 1979 and has maintained a more than 10% annual GDP increase since 1981. There is considerable heterogeneity in economic development within Guangdong Province where the GDP per capita in the most developed municipality is eight times higher than the least developed municipality. Both quantitative national studies [1] and qualitative research [11] have suggested that sexually transmitted infection spread and economic development are related in China. Given the high burden of syphilis and the rapid but uneven economic growth in Guangdong, this provides an excellent opportunity to analyse regional differences in reported syphilis cases. This project analysed the case-based surveillance system (CBSS) of syphilis cases for the province of Guangdong, focusing on changes in primary syphilis cases at the municipal level. The primary objective of this research was to describe the social, demographic, and geographic characteristics of reported primary syphilis cases in order to spatially target syphilis control programs. The secondary objective was to analyse spatial changes in economic development as a single potential determinant of spatial syphilis variation.

## Methods

The Guangdong Provincial STI CBSS has included syphilis as a reportable STI since its inception in 1985. The CBSS system separated primary, secondary, tertiary, congenital and latent syphilis cases starting in 1995. National screening guidelines for the diagnosis of syphilis infection require that each patient receive both a nontreponemal and treponemal test. Although traditional algorithms have involved screening with a nontreponemal test (e.g., TRUST, toluidine red unheated serum test, or rapid plasma reagin test, RPR) followed by confirmation with treponemal test (e.g., TPPA, treponema pallidum particle agglutination), the advent of new rapid syphilis test has allowed initial treponemal testing [12]. Syphilis cases are defined by positive diagnostic tests, history of sexual risk, and characteristic clinical manifestations [13]. Primary and secondary syphilis cases are differentiated based on clinical manifestations, with chancres and buboes suggesting primary syphilis. A confirmed syphilis case requires clinical symptoms consistent with syphilis, a positive treponemal test, and a positive nontreponemal test. The diversity of secondary syphilis clinical manifestations makes this a less sensitive indicator of temporal changes in high risk sexual behaviours, so this study focused on changes in primary syphilis cases.

CBSS data regarding syphilis cases are collected from a number of different types of clinics, including the following: independent STI clinics, general hospital-associated STI clinics, gynecology and obstetrics clinics, family planning clinics, detention centers for sex workers and drug users, and abortion clinics. Guangdong Province has 121 public STI clinics in the province-wide system. There are an average of 5.7 STI clinics in each of the 21 municipalities in Guangdong Province. In each of the clinics, physicians who diagnose syphilis cases are required to fill out a case card with demographic, behavioural, and laboratory data. Case cards are then aggregated by the municipal department and sent to the provincial public health staff where they are entered into the CBSS. The original paper-based CBSS system changed to a web-based, real-time reporting system in 2004. In this system, the individual syphilis cases were entered into the system by each clinic and data components were aggregated by the provincial STI department and reported to respective national and provincial public health authorities. Municipalities were chosen as the level of analysis because data is aggregated at this level and has the most meaningful policy implications.

GDP per capita data at the municipal-level for 2008 was taken from the Guangdong Provincial Yearbook. Spatial analyses were conducted using Legacy GeoDa 0.95i (Tempe, AZ) and maps were constructed using ArcMap (ESRI, Redlands, CA). For spatial analysis, we followed the traditional classification of Guangdong government to divide Guangdong Province into four regions according to the economic development-- the middle region of the province, called the Pearl River Delta, the northern mountain region, the western region and the eastern region. Results were considered significant if the Moran I spatial autocorrelation statistic was less than 0.05. This research study was exempted by the Guangdong Provincial STD Control Center Institutional Review Board in Guangzhou, China.

## Results

**Figure 1:**
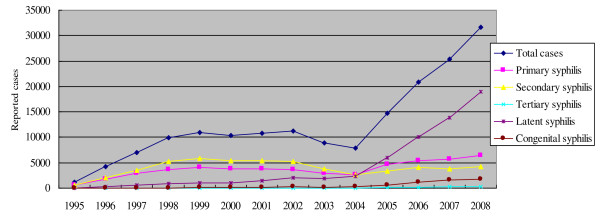
**Guangdong Provincial reported syphilis cases from 1995-2008**.

During the period from 1995 to 2008, the CBSS system reported a total of 174,906 cases of syphilis infection (Figure 1). During this period, primary syphilis cases accounted for 52,036 (29.8%) of the total reported syphilis cases. Primary syphilis burden increased from 0.88 per 100,000 population in 1995 to 5.5 per 100,000 in 1999, representing a six-fold increase in cases. During the period from 1999 to 2003 there was an apparent decrease in primary syphilis cases to 3.74 per 100,000, but the surveillance system was disrupted due to administrative changes. The primary syphilis burden then increased to 7.61 per 100,000 in 2008.

**Figure 2:**
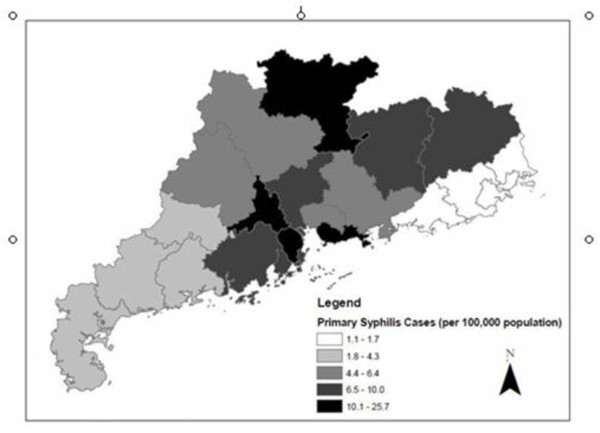
**Municipal-level reported primary syphilis cases in Guangdong Province in 2008**. Source: Guangdong Provincial STD Control Center.

**Figure 3:**
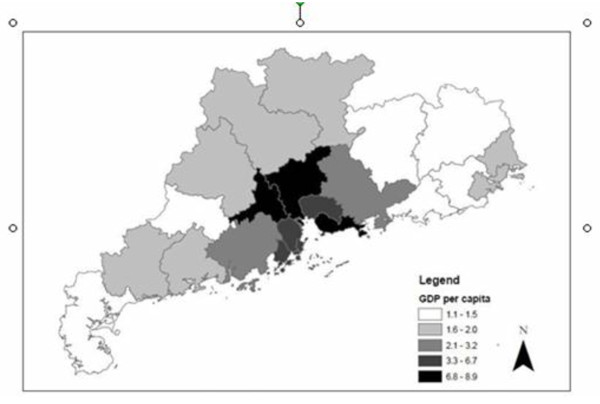
**Municipal-level gross domestic product in Guangdong Province in 2008**. Source: Guangdong 2009 Provincial Yearbook.

Syphilis was reported in all 21 administrative divisions of Guangdong Province. The geographical distribution of syphilis cases in Guangdong Province reveals that syphilis is much more commonly reported in central parts of the province (Figure 2). The middle region of the province, called the Pearl River Delta, has eight municipalities (Guangzhou, Shenzhen, Foshan, Jiangmen, Dongguan, Zhongshan, Huizhou, Zhuhai) that account for 44.7% of primary syphilis cases. The mean primary syphilis cases per 100,000 for the Pearl River Delta region municipalities was 11.4 syphilis cases, followed by the mountain region at 8.9 cases per 100,000 and the western region at 3.6 cases. The lowest burden of primary syphilis cases was in the eastern region with only 1.3 cases per 100,000. The highest syphilis prevalence in 2008 was in the municipality of Zhongshan (25.7 cases per 100,000 population), followed by Foshan (16.2 cases per 100,000 population) and Shenzhen (14.1 cases per 100,000 population). Spatial analysis of syphilis cases in Guangdong Province (including the Pearl River Delta and non-Pearl River Delta regions) aggregated at the municipal-level found significant spatial clustering (Moran's I p-value = 0.012). GDP per capita at the municipal-level was higher in the Pearl Delta Region as well (Figure 3). Multivariate Moran's I analysis of syphilis cases per capita and GDP per capita showed significant spatial clustering (p = 0.004).

**Figure 4:**
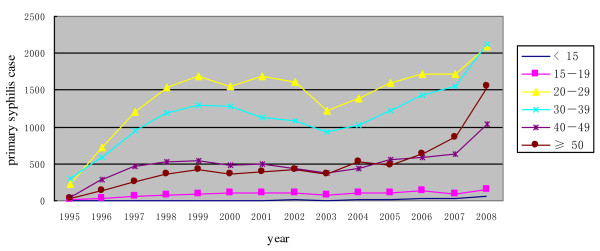
**Primary syphilis cases in Guangdong Province by age group**.

The most rapidly increasing age group affected by syphilis infection was individuals greater than 50 years old (Figure 4). For the past 13 years, the absolute numbers of individuals under 20 years old with syphilis has been stable, with increases in all other age groups (Figure 4). From 2005 on, the most rapidly increasing age group affected by syphilis infection was individuals greater than 50 years old (Figure 4). The male to female ratio of syphilis cases has not changed substantially over the past 13 years. This measure reflects the ratio of male to female syphilis cases included in the reporting system.

**Figure 5:**
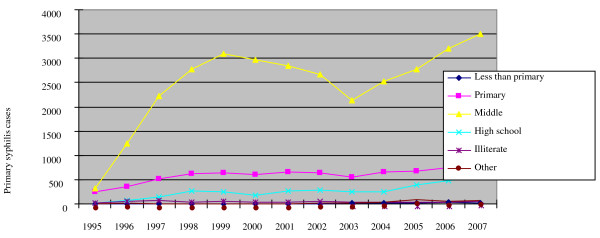
**Education of patients with primary syphilis infection**.

Although data on income is not collected as part of CBSS system, information about the patient's education and occupation is available. 71.6% of patients with primary syphilis infection in 2007 attended middle school, but did not receive further formal education. The group of individuals with only middle school education has increased over the past five years; primary syphilis patients with high school education have also slowly increased over the past five years (Figure 5).

## Discussion

Syphilis is endemic in Guangdong Province, and has made major inroads to its urban municipalities. The single province of Guangdong reported more syphilis cases in 2008 (31,598) than all of the countries in the European Union during the same year [14]. Within China, Guangdong's total number of syphilis cases ranks second among 23 provinces [15]. While there are previous Chinese reviews of reported syphilis cases in Guangdong Province [16-18] this is the first description of changes in primary syphilis cases over time. Changes in reported syphilis cases over time should be interpreted with caution since changes in administrative reporting may account for some major shifts in reported syphilis cases. However, while Guangdong, like much of China [1], had major changes in the syphilis reporting infrastructure in 2003, there have been no administrative syphilis reporting changes since 2003 in Guangdong. The stable syphilis reporting structure and reports of increasing gonorrhea prevalence in Guangdong [15] suggest that the resurgence in primary syphilis is likely related to changes in sexual behaviours.

Earlier work showing that syphilis is more common among developed provinces and municipalities of China [1,2] was confirmed and extended by this analysis. Since the number of reported latent syphilis cases is substantial and more influenced by screening policies than empirical changes in sexual and health seeking behaviour, primary syphilis cases provide important additional information. Other work has shown that individuals in urban China have a higher sexual risk than individuals in rural regions [19]. Higher syphilis case burden in economically developed areas may be related to greater migrants with unsafe sexual behaviours [20], commercial sex industries that do not promote sexual health [21], more communities of homosexual men with unsafe behaviours [22], or a larger number of men with high incomes [19]. Research from Kenya has also found that wealthier individuals have a greater sexual risk [23], another potential pathway between economic development and syphilis infection.

The substantial number of syphilis cases in developed urban regions has two important public health implications: 1) syphilis control measures could be targeted to these geographic regions and may be more cost-effective in these areas [24-26]; 2) syphilis programs focused on individuals in regions with rapid economic development should be considered (e.g., workplace syphilis testing). Although workplace syphilis testing would not reach the whole population, it may be useful to normalize the testing process and reduce stigma associated with seeking clinical sexual health services. In addition, the Global Business Coalition has been active in the fight against HIV in China, and the even closer relationship between economic development and syphilis suggests that there may be a role for business advocacy and involvement in syphilis control programs.

The finding that those older than 50 years old make up an increasing proportion of reported syphilis cases has been noted in national epidemiology data as well [27]. This increase in elder syphilis cases may be due to the aging of China's population, increased availability of medicines for erectile dysfunction, or older man/younger woman commercial sex relations. Our finding that individuals under 20 years old with syphilis have been stable in recent years may have more to do with the low percentage of adolescents who seek sexual health services at public STI clinics [2] than stability in sexual risk among this group. Given that there is a high incidence of syphilis among MSM in China [28-31], a male to female sex ratio among syphilis cases around 1:1 is somewhat surprising. This trend may reflect a growing MSM syphilis epidemic that is being offset by syphilis cases in their female partners, but more data is needed to understand this pattern.

The increasing share of primary syphilis cases among those with limited education reinforces previous similar findings in China [32], underscoring the need for low literacy sex education and interventions. Limited education is also associated with the so-called "surplus men" (poor, young, unmarried men) of China [21], calling attention to how demographic trends may come to influence the spread of sexually transmitted infections [33,34].

There are several limitations to this analysis of reported primary syphilis cases in Guangdong Province. First, only official public clinics report sexually transmitted infections in China, despite the observation that private clinics and unlicensed physicians see a small portion of STI patients in South China [35]. The number of reported syphilis cases is de facto an underestimate based on the large number of clinics that do not routinely report syphilis cases. Second, even among clinics that report syphilis cases, there are likely differences in syphilis screening practices and physician willingness to test STI patients for syphilis [36]. Third, syphilis screening policies in China underwent major changes in 2003 [36]. While mandatory pre-marital syphilis screening was phased out around 2003 in China, many hospitals and clinics have instituted routine screening of hospital inpatients and other populations to help increase case finding. Although our use of primary syphilis cases would limit the influence of increased screening, this trend should not be ignored.

South China faces a severe syphilis epidemic, especially in economically developed areas. Through spatial analysis of primary syphilis cases, we have highlighted some regions that might be useful to target for syphilis testing, treatment, and prevention programs. Such intervention is critical both because of the long term consequences of untreated syphilis as well as the complex but well-established relationship between syphilis and HIV [37,38]. Since a point-of-care rapid test with excellent test characteristics is already available at a range of types of urban clinics in China [35], there is already a foundation to roll-out syphilis screening programs in many areas. Local municipal and province policies for the scale-up of syphilis programs are essential in these regions to ensure comprehensive syphilis control is effective [36].

## Conclusions

South China has a large number of syphilis cases, and the very same municipalities that have high levels of economic growth have been disproportionately affected by syphilis. Based on this connection, drawing support for syphilis control efforts from the broader range of business communities active in Guangdong and promoting syphilis control policies within the framework of economic development may prove to be an effective syphilis control measure.

## Competing interests

The authors declare that they have no competing interests.

## Authors' contributions

YLG, JDT, BY and XSC designed the study and wrote the initial manuscript. YLG, JDT, SYS, XFS did the data analysis. YLG, JDT, BY, YFC and XSC edited the manuscript and completed the final revisions. All authors have read and approved the final manuscript.

## Pre-publication history

The pre-publication history for this paper can be accessed here:

http://www.biomedcentral.com/1471-2458/10/793/prepub
